# Multimodal Cancer Therapy Involving Oncolytic Newcastle Disease Virus, Autologous Immune Cells, and Bi-Specific Antibodies

**DOI:** 10.3389/fonc.2014.00224

**Published:** 2014-09-11

**Authors:** Volker Schirrmacher, Philippe Fournier

**Affiliations:** ^1^Immunologisches Onkologisches Zentrum Köln, Cologne, Germany; ^2^German Cancer Research Institute, Heidelberg, Germany

**Keywords:** Newcastle disease virus, T-cells, dendritic cells, bi-specific antibodies, tumor targeting, cellular therapy, hyperthermia

## Abstract

This paper focuses on oncolytic Newcastle disease virus (NDV). This paper summarizes (i) the peculiarities of this virus as an anti-cancer and immune stimulatory agent and (ii) the approaches to further harness this virus as a vector to combat cancer. Special emphasis is given on combining virus therapy with cell therapy and on improving tumor targeting. The review will include some of the authors work on NDV, bi-specific antibodies, and cell therapy as building blocks for a new perspective of multimodal cancer therapy. The broad anti-tumor immune reactivation includes innate and adaptive, tumor antigen (TA) specific and TA independent activities

## Introduction

Paramyxoviruses are a family of viruses that infect a diverse range of hosts. Animal pathogens, such as Newcastle disease virus (NDV), SV-5, and Sendai virus (SV), have been major subjects for basic research by virologists, immunologists, and molecular biologists. Previously, genetic manipulation of paramyxoviruses was not possible because the genome is not infectious alone and RNA recombination is essentially non-existent. During the last 15 years, methods of producing infectious paramyxoviruses from c-DNA clones (reverse genetics) have been developed. This review will focus on NDV, an avian paramyxovirus, because this has a number of very interesting anti-neoplastic and immune stimulating properties in mammalian cells, including human being, because it has a high safety profile for clinical application and because it can be harnessed by therapeutic transgenes.

## Newcastle Disease Virus, Transgenes, and Bi-Specific Antibodies

### Oncolytic properties of natural strains of NDV

Vaccine strains of paramyxoviruses such as mumps virus (MuV), measles virus (MV), and NDV efficiently infect and kill cancer cells and are consequently being investigated as novel cancer therapies (oncolytic virotherapy) (1). NDV wildtype (wt NDV) virus shows naturally tumor selective replication behavior (2). An abortive replication cycle by lentogenic strains leads eventually to tumor cell death. A lytic replication cycle by mesogenic or velogenic strains leads to fast tumor cell death (oncolysis) and further spread of the virus in the tumor tissue. The strong interferon (IFN) response of normal cells (2) prevents virus replication and cell death thus explaining the high safety record of NDV in cancer patients (3).

There are additional properties that make NDV a particularly interesting anti-neoplastic agent. It replicates and destroys in particular cancer cells that are resistant to certain types of chemotherapy (4–6) and apoptosis-resistant tumor cells from hypoxic tumor tissue (7). The oncogenic protein Rac1 was reported as a link between tumorigenesis and sensitivity of cells to oncolytic NDV (8). Furthermore, NDV triggers autophagy in glioma cells (9) and promotes Bax redistribution to mitochondria and cell death in HeLa cells (10). A time-course analysis revealed that NDV-induced apoptosis involved an early extrinsic pathway with TRAIL expression (peak at 24 h *p.i*.) and a later intrinsic mitochondrial pathway (peak at 48 h *p.i*.) (11).

NDV was reported to repress the activation of human hepatic stellate cells and reverse the development of hepatic fibrosis in mice (12). Liver fibrosis is a major health problem and the 12th most common cause of death in the United States (13).

### Harnessing NDV by transgenes and bi-specific antibodies

Recombinant NDV strains (rNDV) could be harnessed by transgenes to show enhanced oncolytic potential. This was achieved by F gene mutations (14, 15) or by addition of the NS1 (16) or Apoptin (17) gene. It could also be harnessed by genes coding for cytokines, such as IL-2 (18), GM-CSF (19), IL-15 (20), or IFN-γ (21) to express enhanced immune stimulatory properties. Other transgenes conferred resistance to complement (22). NDV was also capable of incorporating two transgenes, one coding for the light chain and the other for the heavy chain of a monoclonal antibody interacting with angiogenesis (23). The transfer of a gene coding for a tumor antigen (TA) created a vector with which the immune response could be targeted to a specific TA in order to compete with the usually stronger response to viral antigens (VA) (24). A recombinant oncolytic MV (MV-AC133) could be targeted to CD133+ cancer-initiating cells causing their specific elimination (25).

To augment the immune stimulatory properties of NDV infected tumor cells, another elegant approach was successful. It consists of the attachment of single-chain variable fragment (scFv) bi-specific antibodies (bsAbs). These attach with one arm to a VA and with the other arm to a target on immune cells. In case of T-cells, such targets were CD3 (26), CD25 (27), and CD28 (28). The VAs of NDV were either HN or F. These served as universal anchor molecules through which T-cell co-stimulatory molecules could be attached to any type of tumor cell infectable by NDV (29).

In the following paragraph, we will present a perspective how such bsAbs can be further used in a multimodal approach for improvements of cancer therapy.

## Future Perspective: Combining NDV with bsAbs and with Adoptive Cellular Therapy

### Tumor targeting of NDV

A major problem with the clinical application of oncolytic viruses is a proper targeting of tumor tissue. This can be achieved by intra-tumoral application (30) but metastases are often not accessible by this approach. Nevertheless, localized oncolytic virotherapy was reported to overcome systemic tumor resistance to immune checkpoint blockade immunotherapy (31).

Locoregional application (e.g., via the hepatic vain) was reported to be superior to systemic tail vain inoculation (32). Locoregional virotherapy was effective even against oncolysis-resistant tumor cells, thus suggesting that the anti-tumor effect was host mediated (32). Inhalation is another way of locoregional application. Inhalation of oncolytic NDV was applied to 33 advanced chemorefractory patients in a Phase II clinical study in Hungary as a means to affect their lung metastases (33). Virus inoculation into body cavities in case of tumor ascites is another way of locoregional application. For instance, intraperitoneal NDV virotherapy was effective against peritoneal carcinomatosis from human gastric cancer in a xenograft model (34) and intrapleural NDV virotherapy induced sustained remission of malignant pleural mesothelioma in an orthotopic model (14).

Upon systemic administration of NDV, its binding to normal cells could prevent it from reaching the tumor tissue and could cause undesired side effects. Since efficient distribution at the tumor site may be a very critical parameter for tumor selective gene delivery and for anti-tumor efficacy of oncolytic virotherapy (35), we have developed adaptor molecules that redirect the virus to tumor tissue (36). The targeting molecule used, anti-HN-IL-2, contains a scFv antibody cloned from a neutralizing HN specific hybridoma linked to the human gene for the cytokine IL-2. Selective virus entry was observed *in vitro* in a mixture of IL-2 receptor positive and negative human tumor cells (37). Retargeted virus infection of tumor cells required specific binding via the bi-specific fusion protein and membrane fusion via the viral F-protein. After systemic virus inoculation into tumor-bearing mice, the modification of NDV by the adaptor protein did not compromise the efficiency of gene delivery into target positive tumors but greatly reduced viral gene expression in target negative tumors and in normal tissues thereby reducing side effects (38).

### Universal activation of cancer patients T-cells (naïve and memory) via tumor cell-bound bi-specific antibodies

**Figure 1:**
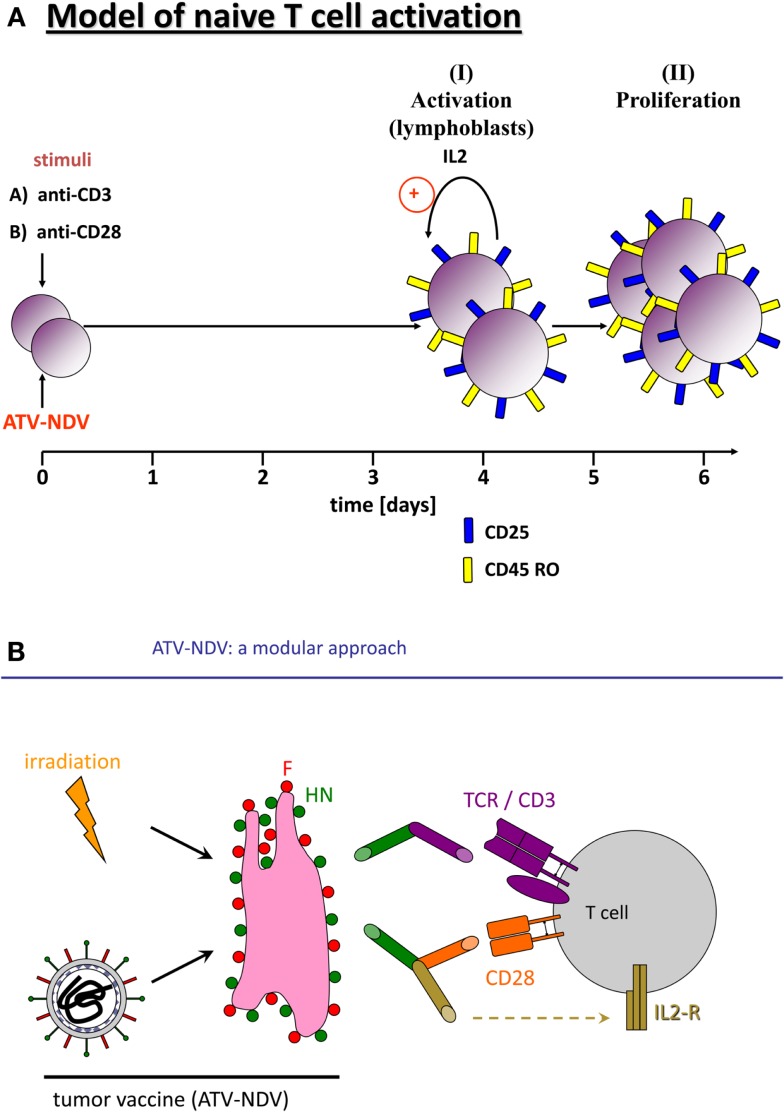
**Activation of naïve human T-cells by co-incubation with NDV infected irradiated tumor cells modified with bi-specific or tri-specific antibodies**. **(A)** Time course of the induction of T-cell activation and proliferation by a stimulatory cell (NDV infected and y-irradiated tumor cells) optimized for co-stimulation by attachment of the bi-specific fusion proteins anti-CD3 (anti-HNxanti-CD3) and anti-CD28 (anti-HNxantiCD28). Purified and CFSE-labeled naïve human T-cells were cocultivated for 5 or 7 days with the stimulatory cells. The CFSE signal intensities were compared with unstimulated cells by FACS analysis. We also followed by the FACS analysis the expression of the IL-2 receptor α chain (CD25) and of the memory marker CD45RO. **(B)** Diagram of the components of a tumor vaccine infected by NDV and modified by a bi-specific antibody (anti-HNxanti-CD3, suboptimal amount for signal 1) and a tri-specific immunocytokine (anti-HNxIL-2xanti-CD28, for delivery of two T-cell co-stimulatory signals via CD28 and CD25).

Infection of tumor cells by NDV leads to increase in tumor cell immunogenicity (39). A prospective, randomized, controlled clinical study of post-operative immunization with the autologous tumor vaccine ATV-NDV revealed evidence for clinical effectivity and long-term survival for colon cancer patients (40). Further augmentation of T-cell stimulatory capacity of the ATV-NDV vaccine was achieved by attachment of specifically designed bsAbs binding to viral HN or F on the infected tumor cells and to CD3 or CD28 on T-cells (41). The optimized vaccine ATV-NDV/bsHNxCD3/bsHNxCD28 appeared to be able to revert unresponsiveness of partially anergized TA-specific T-cells (42). It was also capable of *de novo* activation of anti-tumor activity from naïve T-cells, independent of TA recognition (Figure 1A) (42). The strongest potentiation of the T-cell stimulatory capacity of the ATV-NDV vaccine was observed upon attachment of a suboptimal amount of bsHNCD3 together with the tri-specific (ts) fusion protein tsHNxIL-2xCD28. The latter delivers two co-stimulatory signals to T-cells, one via CD28 and the other via CD25 (26). Figure 1B illustrates the modular concept of the tumor vaccine infected by NDV and modified by bsAbs.

We suggest to use T-cell activation one universal GMP tumor cell line for patients. This will be modified by infection with NDV and by attachment of the above bsAbs and tsAbs. This universal T-cell stimulatory cell can be applied for non-specific activation of anti-tumor activity of T-cells from any type of cancer patient and is independent from a TA.

### Programing of cancer patients dendritic cells toward DC1 via infection by NDV

We reported on polarization of human monocyte-derived DCs to DC1 by *in vitro* stimulation with NDV (43). Also, murine DCs upon infection by NDV differentiate into the immunogenic phenotype DC1 characterized by secretion of pro-inflammatory cytokines, in particular IL-12 and IFN-α and -β (44). Two receptor-initiated signaling cascades were involved: the first one is induced by triggering and upregulation of the intra-cellular cytoplasmic receptor RIG-1 upon recognition of viral non-capped RNA as ligand (45). The second signal cascade involves cell-surface expressed type I IFN receptor (IFNAR), which initiates a feedback loop cell activation upon interaction with extra-cellular type I IFN as ligand (31, 44). RIG-1/RNA ligand interaction not only activates type I IFN, but also induces inflammasome activation for IL-1β production (46). Type I IFN and IL-12 are critical mediators of cross-priming and Th1 polarization of CD8 T-cell responses (47) while IL-1β is critical for Th1 polarization of CD4 T-cells (48).

DCs can also be pulsed with NDV oncolysate. Such cells were superior in stimulating patients T-cells in ELISPOT assays compared to DCs pulsed with tumor lysate without NDV (49).

### Grafting of autologous activated T-cells and DC1 back to the patient

Our proposal for a multimodal cancer therapy involves the transfer of immune T-cells and of DC1 as professional antigen-presenting cells back to the patient. Activation of the tumor microenvironment by low dose irradiation (LDI) (50) or by local hyperthermia (LHT) (51) should improve tumor targeting of virus, T-cells, and DCs (52). Tumor destruction by the activated T-cells should release TAs, which would be taken up by co-injected DC1 to be then cross-presented to naïve or memory T-cells.

### Hitchhiking of NDV on activated T-cells: Combining cell therapy with virus therapy

One way of further enhancement of the efficacy of this multimodal therapy concept consists in the loading of the activated T-cells with oncolytic NDV before grafting the cells back to the patient. In a tumor neutralization assay *in vitro*, monolayers of human tumor cells could be completely and effectively destroyed by the addition of polyclonally activated human T-cells loaded with oncolytic NDV (53). In this process, synergistic effects between cytotoxic T-cells and oncolytic virus in the tumor contact zone were apparent (53).

If activated T-cells are not available, a multimodal therapy could also consist of the combination of LHT, systemic application of oncolytic NDV and of DC1. Such approach resulted in long-term remission of metastatic prostate cancer (52).

### Targeting an introduced viral antigen in tumor tissue by grafted T-cells and DCs via cell-bound tri-specific antibodies

Table 1 summarizes five steps that are essential for a new adoptive cellular cancer therapy strategy. Oncolytic NDV can be introduced into tumor tissue of the patient by various means as discussed before. The patients T-cells and DCs would be activated and polarized also as discussed before. The tsAbs have three different binding sites, each of which is only monovalent. To increase the avidity and stability of the cell surface attached ts fusion protein, we propose that two of the binding sites should bind to well-defined targets on T-cells or DCs. The addendum of the table lists some of the potential targets.

**Table 1:** **Adoptive cellular cancer therapy: targeting a viral antigen (e.g., HN) by grafted T-cells and DCs via cell-bound tri-specific antibodies**.

Step 1	Pre-conditioning of the tumor microenvironment in the patient
Step 2	Local or systemic application of oncolytic NDV for introduction of the viral target antigen HN within the tumor tissue
Step 3	Universal activation ex vivo of the patients T-cells and loading with tri-specific antibodies thus exposing multiple anti-HN binding sites
Step 4	Generation of polarized DCs from the patient via infection by NDV or pulsing with NDV oncolysate; loading of the DC1 with tri-specific antibodies thus exposing multiple anti-HN binding sites
Step 5	Grafting the T-cells and/or DCs to the pre-conditioned patient

***Addendum*:** The tri-specific single-chain antibodies should bind with two arms to targets on T cells or targets on DCs and expose the third arm anti-HN	

**Potential T-cell targets**	**Potential DC targets**

CD3	CD11c
CD28	CD205
CD25	CD40
CD2	CD80
CD44	CD16a
CD45	CD83
CD69	CD116
CXCR4	IFNAR
CD107a	CD119

This approach is only meant as a perspective for the future and has not been tested experimentally or clinically. There should be a proper timing between virus-pretargeting of tumor tissue (including metastases) and the cell therapy. We envisage that 24–48 h after virus inoculation should be a good time period for grafting the cells for a VA targeted therapy. Excessive virus should be cleared by then and the tumor tissue should be infected and expressing cell-bound VAs.

## Concluding Remarks

We propose a multimodal approach for effective cancer therapy because previous monomodal approaches of chemo- or radiotherapy faced problems of tumor resistance mechanisms. Specific immunotherapies targeted to specific TA faced similar problems of tumor escape and resistance mechanism. There may be a long way to get a multimodal therapy such as the one proposed and established but we believe it is important to propose a viable perspective for future orientation. Oncolytic viruses, T-cells, dendritic cells, and bi-specific antibodies are all promising biologics whose intelligent combination holds a lot of promise for future cancer therapy.

## Conflict of Interest Statement

The authors declare that the research was conducted in the absence of any commercial or financial relationships that could be construed as a potential conflict of interest.
